# 331. Hospitalized COVID-19 infections in Infants < 1 Year of Age

**DOI:** 10.1093/ofid/ofab466.533

**Published:** 2021-12-04

**Authors:** Christina Gagliardo, Eberechi I Nwaobasi-Iwuh, Niva Shah, Aparna Prasad, Neeraja Kairam, Stephanie Chiu, Elizabeth DeSantis, Simona Nativ, Kathryn Laurie, M Cecilia Di Pentima

**Affiliations:** 1 AHS/TJU, Morristown, New Jersey; 2 AHS, Springfield, New Jersey

## Abstract

**Background:**

Nearly 4 million children have tested positive for coronavirus disease 2019 (COVID-19) in the United States. Some studies suggest infants might be at increased risk for severe illness and hospitalization from COVID-19. Our objective was to describe the clinical and laboratory features of young infants admitted to a hospital system with COVID-19.

**Methods:**

An observational retrospective study was performed in infants ≤1 year of age admitted with COVID-19 from March 1, 2020 to May 30, 2021. Data was extracted into a REDCap database and analyzed using descriptive statistics.

**Results:**

Sixteen infants < 1 year were hospitalized with COVID-19. Fever, poor feeding, and respiratory symptoms were the most common presenting symptoms (Table 1). Two required pediatric intensive care unit (ICU) care, three required oxygen support, and one was intubated. There were no deaths. Five infants with echocardiograms performed showed normal findings. Four infants received Remdesivir without side effects.

**Conclusion:**

Infants with COVID-19 can present with severe disease requiring ICU care and oxygen support. In our experience, a large proportion of infants developed hematologic abnormalities, but none had cardiac involvement. Preventive measures including vaccination will become critical to decrease transmission and severe disease in this young patient population.

**Disclosures:**

**All Authors**: No reported disclosures

**Table:** 

Characteristics	Patients (N=16)
Age, range	2 weeks to 10 months
Male, n (%)	10 (63)
Hispanic, n (%)	7 (44)
Race	
Black n (%)	1 (6)
White n (%)	10 (63)
Prematurity n (%)	3 (19)
Comorbid condition, n (%)	0
Contact with COVID-19 positive individual n (%)	13 (81)
**Clinical presentation**	
Fever n (%)	13 (81)
Cough n (%)	5 (31)
Rhinorrhea n (%)	5 (31)
Respiratory distress n (%)	2 (12)
Poor feeding n (%)	5 (31)
Emesis n (%)	3 (19)
Diarrhea n (%)	2 (13)
Hypoxia n (%)	3 (19)
**Laboratory**	
Absolute neutrophil count, median (min, max)	0.64 (0, 1.16)
Neutropenia (< 1.5) n (%)	7 (44)
Lymphopenia (< 4.5) n (%)	12 (75)
Thrombocytopenia n (%)	3 (19)
C-reactive protein highest value, mg/L, median (IQR)	14. 3 (2.33, 11.73)
**Disposition & Therapies**	
Length of hospital stay, days, median (range)	4.5 (1, 16)
ICU admission n (%)	2 (13)
Any oxygen support, n (%)	3 (19)
Mechanical ventilation, n (%)	1 (6)
Vasopressor support n (%)	0
Antibiotics, n (%)	13 (81)
Remdesivir n (%)	4 (25)
Steroids, n(%)	1 (6)

